# Society Meetings

**Published:** 1902-12

**Authors:** 


### SOCIETY MEETINGS.

The Southern Surgical and Gynecological Association held its fifteenth annual meeting at Cincinnati, November 11-13, 1902, under the presidency of Dr. William E. B. Davis, of Birmingham. It was an unusually interesting occasion, the attendance being large, the papers and discussions spirited and the weather propitious. This association is doing most excellent work and deserves, as it receives, the support of some of the ablest medical men in the country.

The social features of the meeting were especially attractive. They included a luncheon by Dr. Joseph Ransohoff on Tuesday, at the Phoenix Club, which was attended by a large number of members and guests; dinners on Tuesday evening by Drs. Rufus B. Hall, N. P. Dandridge and Charles A. L. Reed, at the Queen City Club; a general luncheon for the entire association and guests at the Grand Hotel, on Wednesday, given by the medical profession of Cincinnati; a banquet on Wednesday evening at the Queen City Club, also by the Cincinnati profession, and a luncheon on Thursday, given by Drs. Edwin and Merrill B. Ricketts. The banquet was one of the best medical dinners ever given to a society. The toastmaster, Dr. Dan Milliken, of Dayton, resplendent with eloquence, wit, humor and general good nature, made the occasion one long to be remembered.

Dr. J. Wesley Bovée, of Washington, was elected president; Drs. Bacon Saunders, of Fort Worth, Tex., and Christopher Tompkins, of Richmond, Va., vice-presidents, and the next meeting was appointed to be held at Birmingham in November, 1903.

---

THE American Public Health Association will hold its thirtieth annual meeting at New Orleans, Monday, Tuesday, Wednesday, Thursday and Friday, December 8, 9, 10, 11, and 12, 1902.

The executive committee has selected the following topics for consideration, but papers will be received upon other sanitary subjects.

General meeting—(1) The pollution of public water supplies; (2) The disposal of refuse material; (3) Animal diseases and animal food; (4) Car, steamship and steamboat sanitation; (5) Etiology of yellow fever; (6) Demography and statistics in their sanitary relation; (7) Cause, prevention, period of incubation and duration of infectious diseases; (8) Public health legislation; (9) Cause and prevention of infant mortality; (10) Disinfectants and disinfection; (11) National leper homes; (12) Dangers to the public health from illuminating and fuel gas; (13) Transportation of diseased tissue by mail; (14) The teaching of hygiene and granting of diploma of doctor of public health; (15) Sanitary aid societies; (16) The relative immunising value of human and bovine vaccine virus; (17) The investigation of the canteen system of the United States Army.

Dr. W. D. Greene, Health Commissioner, and Dr. W. G. Bissell, bacteriologist of the Department of Health, will attend the meeting from Buffalo.

---

The Western Surgical and Gynecological Association will hold its twelfth annual meeting at St. Joseph, Mo., December 29 and 30, 1902, under the presidency of Dr. James E. Moore, of Minneapolis. Dr. George H. Simmons, Chicago, is the secretary, and Dr. C. H. Wallace, St. Joseph, is chairman of the committee of arrangements.

---

The American Röntgen Ray Society will hold its third annual meeting at Chicago, December 10-11, 1902, under the presidency of Dr. G. P. Girwood, of Montreal. The sessions of the society, as well as the exhibits, will be held in the Sherman House, Randolph and Clark Streets. Dr. Ralph R. Campbell is chairman of the local committee of arrangements.

Owing to the fact that papers which are to be read at this meeting have to be submitted to the executive committee for endorsement before being placed on the program, it is found impossible this year to issue a preliminary program, but assurance is given that the names which will appear on the program are of the best *x*-ray workers in the country and that the matter will be unexceptionable.

For further information address the secretary, James B. Bullitt, 205 W. Broadway, Louisville, Ky.

---

The Mississippi Valley Medical Association held its 28th annual meeting at Kansas City, October 15, 16 and 17, 1902, when the following-named officers were elected for the ensuing year; president, Edwin Walker, Evansville; first vice-president, Hugh T. Patrick, Chicago; second vice-president, Wm. Britt Burns, Memphis; secretary, Henry Enos Tuley (re-elected), Louisville; treasurer, Thos. Hunt Stucky (re-elected), Louisville. The next annual meeting will be at Memphis, October 7, 8 and 9, 1903.

---

The Medical Association of Central New York held its thirty-fifth annual meeting at Syracuse, N. Y., October 21, 1902, under the presidency of Dr. A. L. Beahan, of Canandaigua. The following-named officers were elected for the ensuing year: president, E. L. Heffron, Syracuse; first vice-president, C. A. Van der Beek, Rochester; second vice-president, M. L. Bennett,

Watkins; secretary, C. A. Greenleaf, Rochester, and treasurer, William M. Brown, Rochester. The next annual meeting will be held at Auburn in October, 1903. A full report of the proceedings will be found on page 347.

---

THE Buffalo General Hospital Alumni Association was organised at the Hotel Niagara on a recent date. It is composed of the ex-internes of the Buffalo General Hospital and after a banquet the following-named officers were elected: president, Dr. D. W. Harrington; vice-president, Dr. P. W. Van Peyma; secretary-treasurer, Dr. Wm. H. Thornton. Executive committee: Drs. Thomas B. Carpenter, Edgar R. McGuire, and Edwin R. Gould, Buffalo.

---

THE New York State Medical Association at its annual meeting held at New York, October 20-24, 1902, elected the following-named officers: president, Frederick Holme Wiggin, New York; vice-president, William H. Thornton, Buffalo; secretary, Guy Davenport Lombard, New York; treasurer, E. H. Squibb, Brooklyn; for chairmen of committees: arrangements, Samuel A. Brown, New York library, John Shrady, New York; legislation, E. Eliot Harris, New York; public health, John Scott Wood, Brooklyn; publications, Emil Mayer, New York; nominations, Charles E. Quimby, New York; for delegates to the American Medical Association for two years, Joseph D. Bryant, New York; Elias Lester, Seneca Falls; alternates, Parker Syms, New York; L. D. Farnham, Binghamton.

---

THE sixth meeting of the Congress of American Physicians and Surgeons will be held in Washington, D. C., May 12, 13 and 14, 1903, under the presidency of Dr. William W. Keen, Philadelphia.

Program: The meeting of the congress will be held in the Columbia Theatre, corner of 12th and F Streets. Tuesday, May 12, 3 p.m., the congress will be opened by the president. Subject to be considered: The pancreas and pancreatic diseases. Papers will be read as follows: E. L. Opie, Baltimore, on the anatomy and histology; Prof. R. H. Chittenden, New Haven, on the physiology and physiological chemistry; Simon Flexner, Philadelphia, on the etiology and pathological anatomy; Reginald H. Fitz, Boston, on the symptomatology and diagnosis; Prof. von Mikulicz, Breslau, Germany, and Roswell Park, Buffalo, on the surgery. Followed by a discussion by Charles

G. Stockton, Herbert U. Williams and Maurice H. Richardson. At 8 p.m., address by the president of the congress, William W. Keen, professor of surgery in the Jefferson Medical College, on The duties and responsibilities of trustees of medical institutions.

Wednesday, May 13, 3 p.m., subject to be considered: The medical and surgical aspects of the diseases of the gall-bladder and bile ducts. Papers will be read as follows: Prof. Ewald, Berlin, Germany, John H. Musser, Philadelphia, C. A. Herter, New York, on the pathology and therapy; Prof. Hans Kehr, Halberstadt, Germany, A review of eight hundred cases of gall-stone operations. William J. Mayo, Rochester, Minn., George E. Brewer, New York; on the surgery. Followed by a discussion by Frank Billings, George Dock, W. S. Halsted and Henry Sewall.

Thursday evening, May 14, 8 p.m., "Smoker" at the New Willard Hotel, corner of 14th Street and Pennsylvania Avenue.

---

THE Buffalo Academy of Medicine held meetings during the months of October and November, 1902, as follows:

*Section on Surgery.*—Tuesday evening, October 7. Program: A summary of thirteen operative cases on the gall-bladder and ducts, Eugene A. Smith.

*Section on Medicine.*—Tuesday evening; October 14. Program: The works of Edward Jenner, and their value in the modern study of smallpox, George A. Dock, Ann Arbor, Mich.

Tuesday evening, November 11. Program: Medical discoveries by non-medical men, George M Gould, Philadelphia; How epileptics are cared for and treated at Sonyea, William P. Spratling, superintendent of Craig Epileptic Colony, Sonyea.

*Section on Pathology.*—Tuesday evening, October 21. Program: A case of unusual injury of head, recovery, subsequent autopsy, findings, Henry P. Frost; Eosinophilia in trichinosis. Report of a case and review of the literature, Edwin R. Gould.

Tuesday evening, November 18. Program: The pathology and sanitary significance of bovine tuberculosis, Prof. Veranus A. Moore, Cornell University, Ithaca.

*Section on Obstetrics and Gynecology.*—Tuesday evening, October 28. Program: Report of case of extreme marasmus with recovery, D. E. Wheeler; Eclampsia, John V. Woodruff; Infant feeding, A. J. Colton.

Tuesday evening, November 25. Program: Pelvic abscess and its treatment, Herman E. Hayd; Period of dentition, H. Singer.

*Stated Meeting.*—Monday, November 3. Program: The physics and therapeutic value of the (Röntgen) cathode and ultra-violet rays, with certain references to the electro-magnetic theory of light; with exhibition of new instruments for phototherapy, Roswell Park.

